# The development and trial of a medication discontinuation program in the department of forensic psychiatry

**DOI:** 10.1186/s12991-015-0049-z

**Published:** 2015-02-27

**Authors:** Kenji Murasugi, Teruomi Tsukahara, Shinsuke Washizuka

**Affiliations:** Department of Psychiatry, National Hospital Organization, Komoro Kogen Hospital, 4598 Kou, Komoro, Nagano 384-8540 Japan; Department of Psychiatry, Shinshu University School of Medicine, Nagano, Japan; Department of Preventive Medicine and Public Health, Shinshu University School of Medicine, Nagano, Japan

**Keywords:** Medical Treatment and Supervision Act, Adherence, Medication Discontinuation Program, Schizophrenia, Drug Attitude Inventory-30

## Abstract

**Background:**

When treating mentally ill criminal offenders, improving medication adherence is essential to achieving goals, such as long-term stabilization of symptoms and the prevention of recidivism. Most subjects who are treated under the Medical Treatment and Supervision Act have schizophrenia, which is considered a particularly difficult disorder for which to improve medication adherence. For such patients, we developed a Medication Discontinuation Program (MDP) that aims to improve medication adherence by discontinuing antipsychotic drugs and monitoring changes in psychiatric symptoms. We examined whether there was any utility for the MDP on a trial basis as well as whether it would be worthwhile to introduce the MDP to psychiatric programs.

**Methods:**

We conducted the MDP with an intervention group (*n* = 7) and compared Drug Attitude Inventory-30 (DAI-30) scores before and after implementation of the MDP. We also categorized 30 questions of the DAI-30 into three subscales: “awareness of the need for medication”, “awareness of the effects of psychiatric drugs”, and “impression of medication”, and examined factors affecting improvement in medication adherence.

**Results:**

The total DAI-30 score significantly increased after completion of the MDP (*P* = 0.002). Significant elevations after completion of the MDP were also observed in the scores for three subscales of the DAI-30.

**Conclusions:**

Our study suggests that the MDP has a possibility of improving medication adherence, and this program might have multidirectional and stimulatory effects on each factor related to the improvement of medication adherence.

## Background

**Figure 1:**
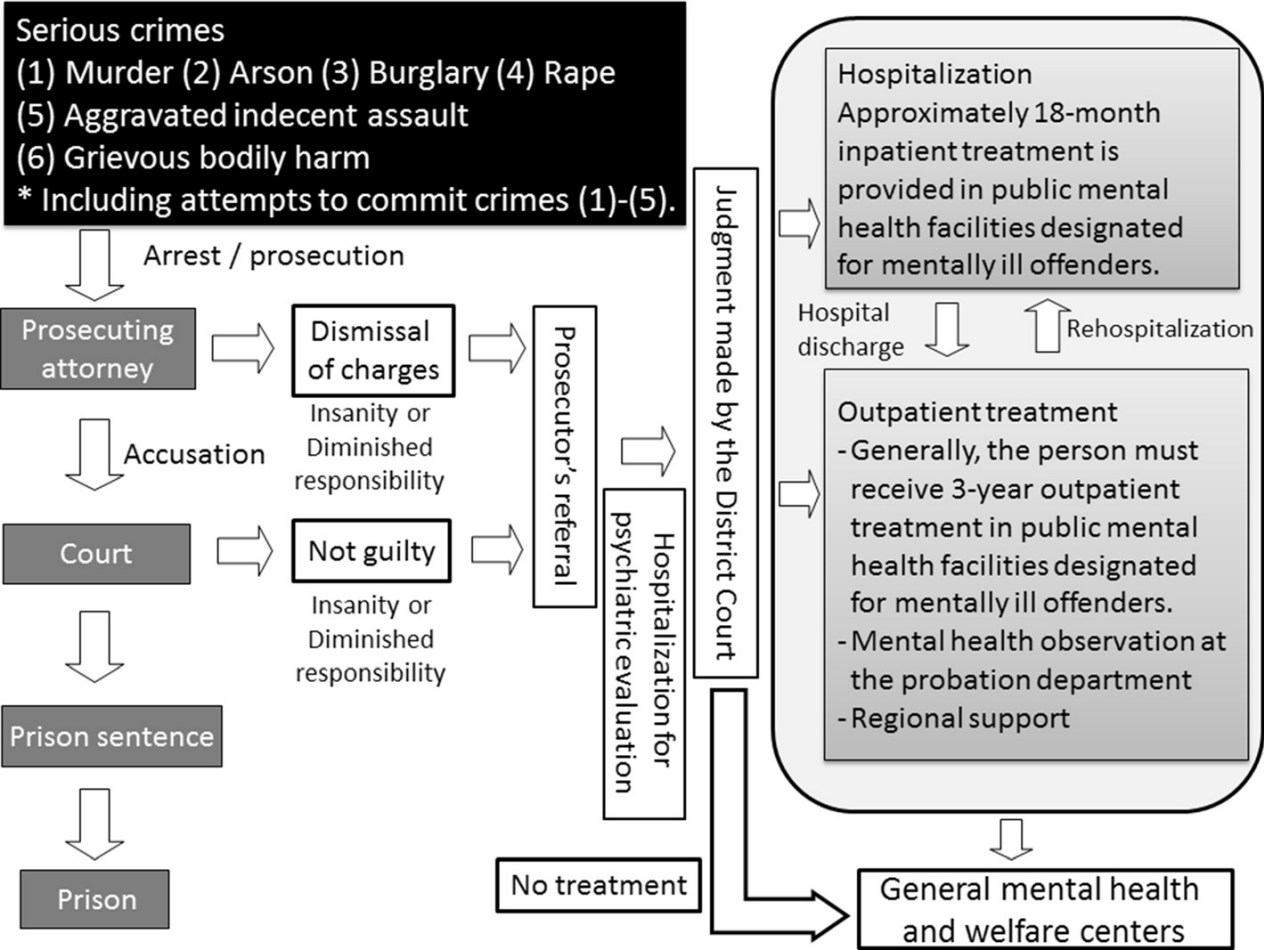
**Framework of the Medical Treatment and Supervision Act (MTSA).**

The preparation of special treatment systems and facilities is important so that mentally ill people who have committed crimes can be provided with appropriate treatment and avoid recidivism. However, no such system was in place in the late 19th century when scientific medicine was introduced to Japan. More surprisingly, no system was developed in Japan until the early 21st century. Forensic support services in Japan lag far behind those in European countries and the United States, and recently, this need was recognized. As a result, the Act on Medical Care and Treatment for Persons Who Have Caused Serious Cases Under the Condition of Insanity (hereafter, the Medical Treatment and Supervision Act (MTSA)) was signed into law on 15 July, 2005, as the first law pertaining to the treatment and social reintegration of offenders with mental illness in Japan. The purpose of the MTSA is to promote the social rehabilitation of people who have committed serious criminal offenses due to mental disorders. To achieve this goal, subjects are provided with appropriate medical care and continuous supervision to improve their psychiatric symptoms and prevent their recidivism. The framework of the MTSA is shown in Figure 1, and details have been reported previously [1].

**Figure 2:**
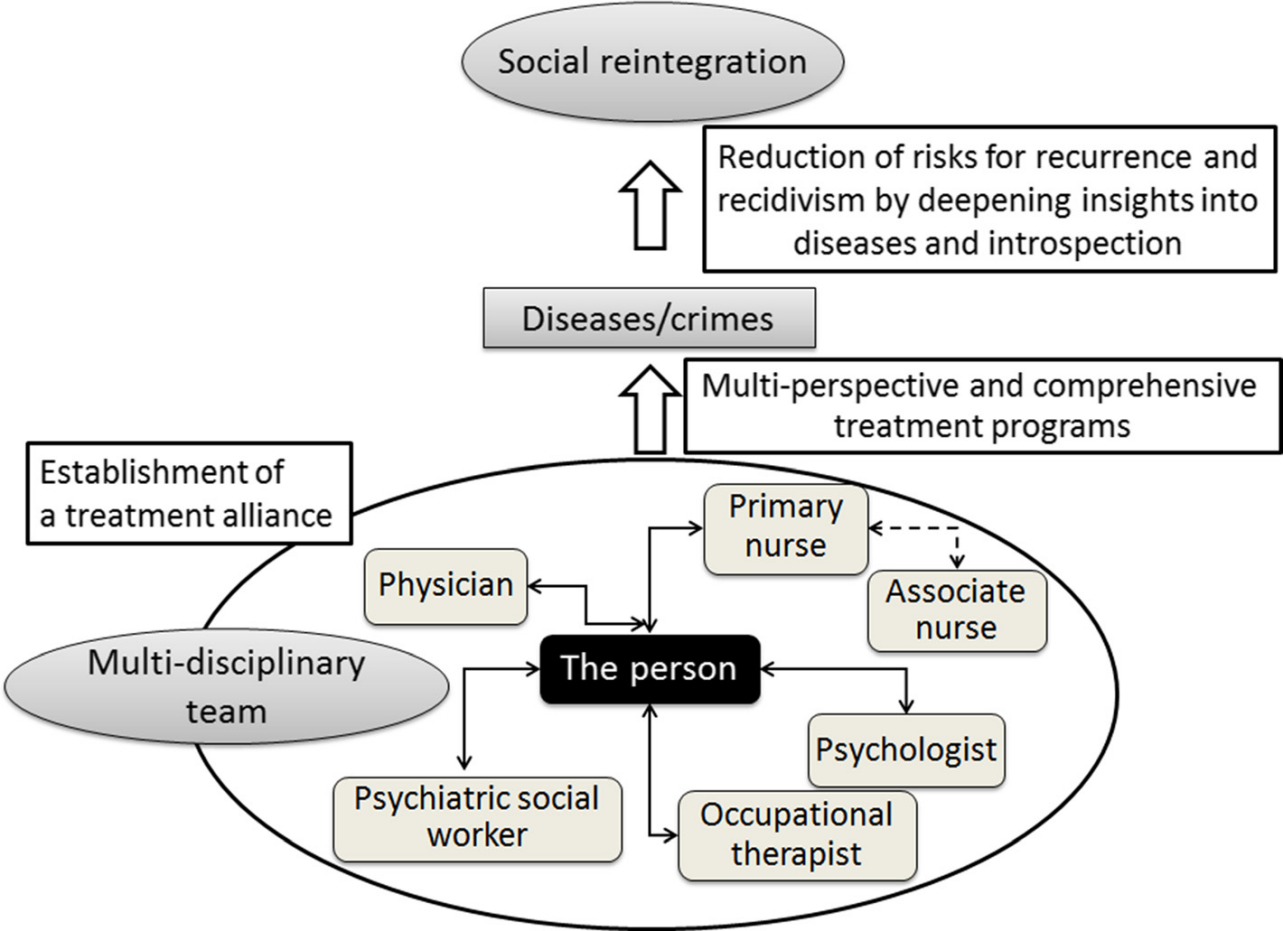
**Structure of treatment provided under the Medical Treatment and Supervision Act (MTSA).**

Psychiatric treatment provided under the MTSA aims to achieve the goals of the MTSA by (1) building a treatment alliance between a person who is subject to the MTSA and a multidisciplinary team consisting of a psychiatrist, psychologist, nurse, occupational therapist, and psychiatric social worker; (2) helping the patient recognize the presence of a mental disorder and take responsibility for crimes caused by the illness; and (3) deepening insight and introspection into the disease (Figure 2).

**Figure 3:**
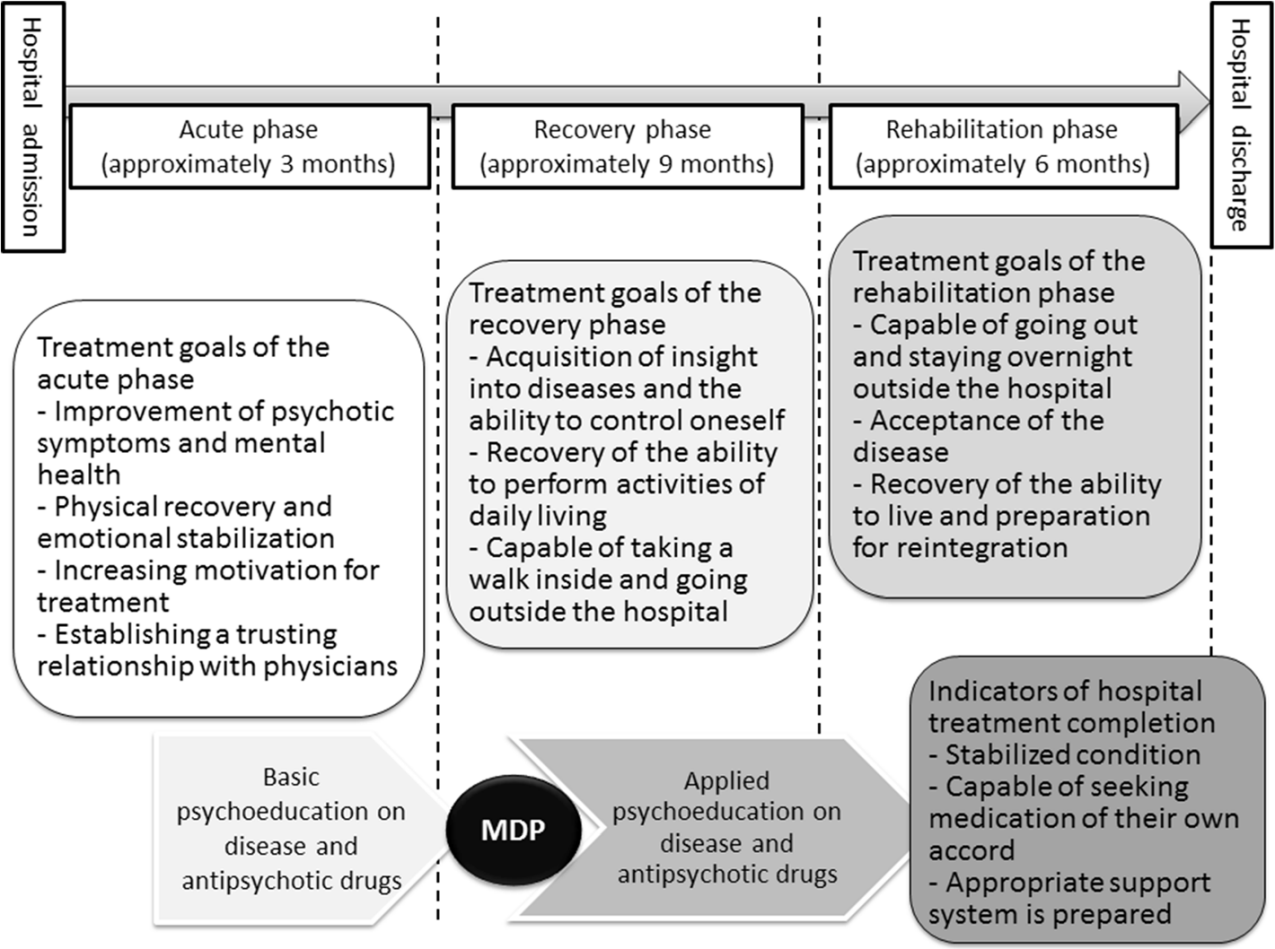
**Process of hospital treatment provided under the Medical Treatment and Supervision Act (MTSA).** The Medication Discontinuation Program (MDP) was conducted in the early stages of the recovery phase, in which medical conditions were stabilized.

Guidelines for psychiatric treatment provided under the MTSA published by the Japanese Ministry of Health, Labour and Welfare offer an approximately 18-month hospitalization, which is divided into three treatment phases: acute, recovery, and rehabilitation. Each phase has the following main achievement goals: improving symptoms, enhancing motivation in treatment, and establishing trust relationships with the persons who committed crimes in the acute phase; acquiring insight into diseases and the ability to control oneself and improving medication adherence in the recovery phase; and recovering the ability to live and preparing for reintegration in the rehabilitation phase (Figure 3). The improvement in medical adherence is an extremely important treatment issue for achieving treatment goals and social reintegration. Some reports show that denial of medication is an important predictor of violence derived from psychotic symptoms [2,3]. Moreover, Swartz et al. reported that drug abuse combined with poor adherence to medication among inpatients with severe mental illness may indicate a higher risk for violent behavior in the community after discharge [4].

Most people who are treated under the MTSA have schizophrenia [5]. Full medication adherence is reported to be rare in schizophrenia compared with that in other diseases [6]. The following factors are considered to be associated with medication adherence: patient-related factors [7-9], such as levels of insight, psychotic symptoms, cognitive impairment, education level, disease duration, and adaptation to society; medication-related factors [10-12], such as the presence or absence of side effects and dosing frequency; and environmental factors [9,12-14], such as the patient-physician relationship, family support, and financial status.

To improve medication adherence, we provide disease education and prescribing information along with single atypical antipsychotic therapy, and many patients have achieved social reintegration after receiving interventions to improve medication adherence. However, there were some patients who continue to deny the presence of their illness and reject treatment because of poor insight and lack of understanding of the need to take medication. As far as we know, no studies in Japan or European countries and the United States, the latter two of which have a longer history of offering specialized services and facilities for mentally ill offenders, have reported an effective method to improve medication adherence for patients refusing to take medication.

Therefore, we conducted a Medication Discontinuation Program (MDP), which was developed to increase awareness of the effects of psychiatric drugs and improve patients’ insights into their own disease and medication adherence. The MDP accomplishes this goal by having patients discontinue their medication and then track the change in their behaviors when not medicated. After seeing these changes, it is hoped that patients will recognize the importance of adhering to their medication regimen in the future to maintain more appropriate behavior. The program was performed between patients and a multidisciplinary team who worked to discontinue antipsychotic drugs using an individually structured approach and monitor changes in psychiatric symptoms. To examine whether there was any utility for the MDP, we conducted the MDP with seven patients with schizophrenia (intervention group) and compared Drug Attitude Inventory-30 (DAI-30) scores before and after implementation of the program [15]. The DAI-30 comprehensively estimates subjective reactions and attitudes to antipsychotic drugs in patients with schizophrenia. We also examined factors that contribute to improving medication adherence.

## Methods

### Subjects

The present study was conducted for inpatients in the Department of Forensic Psychiatry at Komoro Kogen Hospital from March 2009 to August 2013. Subjects undergoing the MDP were selected by experienced psychiatrists who based their assessments on the Diagnostic and Statistical Manual of Mental Disorders - Fourth Edition (DSM-IV) [16]. Subject selection criteria were as follows: among patients who were diagnosed as having schizophrenia, those who did not show improvement in medication adherence during standard treatment, including single atypical antipsychotic therapy, psychoeducation on disease, and neuroleptics, and who exhibited strong denial of their disease and medication refusal. For subjects to monitor themselves and verbally express their own condition after discontinuation of medication, this study also specified the following inclusion criteria: subjects had to have scored more than 70 on the full-scale Wechsler Adult Intelligence Scale - Third Edition (WAIS-III) and more than 41 on the Global Assessment of Functioning (GAF), which indicates that the patient has a moderate level of communication skills and no tendency to hide their condition. A total of eight cases with schizophrenia who had committed crimes due to persecutory delusions met the above criteria (Table 1). The age, gender, and crime of each participant are not shown to maintain anonymity of participants. Case 2 was excluded from the study because he did not reach the acceptable level of medication readministration criteria after discontinuation of antipsychotic drugs. The criteria used in the MDP provide a standard description at which the subject has to resume taking antipsychotic drugs according to the deterioration of psychotic symptoms (Table 2). The remaining seven cases (the intervention group) were included in this study.

**Table 1:** **Characteristics of subjects undergoing the Medication Discontinuation Program (MDP)**

	**Case** ^**a**^	**DUP (year)**	**Treatment duration (year)**	**Academic achievement**	**WAIS-III full- scale IQ**	**Experience of medication discontinuation**	**Family members living together**	**GAF score**	**Main antipsychotic drug**	**CP equivalent (mg)**
Intervention group	Case 1	20	9.66	Graduate school	95	Present	Absent	42	OLZ	300
	Case 2^b^	2	0.58	Graduate school	110	Absent	Absent	51	QTP	1,060.6
	Case 3	16	0.25	Graduate school	114	Absent	Absent	55	OLZ	200
	Case 4	0.42	0.42	Undergraduate school	73	Present	Present	51	RIS	600
	Case 5	5	9.58	High school	88	Present	Present	45	OLZ	600
	Case 6	6	0.67	High school	89	Present	Present	56	OLZ	300
	Case 7	4	0.25	Undergraduate school	106	Absent	Absent	63	RIS	200
	Case 8	1	0.25	High school	77	Absent	Present	55	OLZ	700
Non-intervention group	Case a	2	0.67	High school	87	Present	Present	58	OLZ	400
	Case b	0.5	5	Undergraduate school	104	Present	Present	42	RIS	1,000
	Case c	1.67	7.33	Undergraduate school	122	Present	Present	60	RIS	400
	Case d	0.33	25.5	Undergraduate school	108	Present	Present	61	OLZ	200
	Case e	19.33	1	Graduate school	85	Present	Present	33	OLZ	1,200
	Case f	0.08	24.75	Undergraduate school	93	Present	Present	53	OLZ	1,000
	Case g	5	36.58	Junior high school	71	Present	Absent	35	OLZ	800
	Case h	1.41	11.33	High school	90	Present	Present	42	QTP	2,218.2
	Case i	4.25	0.58	High school	101	Absent	Present	55	OLZ	650
	Case j	5	0.33	High school	70	Absent	Present	45	ARP	150
	Case k	0.33	1.91	Graduate school	98	Present	Present	53	OLZ	600
	Case l	0.16	11.75	Undergraduate school	100	Present	Absent	63	QTP	757.6
	Case m	0.04	1.25	High school	85	Present	Present	68	OLZ	100
	Case n	5	0.25	Undergraduate school	78	Absent	Present	62	QTP	606.06
	Case o	5	0.16	Undergraduate school	114	Present	Absent	55	OLZ	600
	Case p	4	4	Undergraduate school	100	Present	Present	61	RIS	400
	Case q	0.08	34.75	High school	72	Present	Present	57	OLZ	800

^a^Numbered cases underwent the intervention; lettered cases did not.

^b^Case 2 was excluded from this study because he did not reach an acceptable level of the medication readministration criteria after discontinuation of antipsychotic drugs and a failure to restart them.

*DUP* duration of untreated psychosis, *WAIS-III* Wechsler Adult Intelligence Scale - Third Edition, *GAF* Global Assessment of Functioning, *CP* chlorpromazine, *OLZ* olanzapine, *QTP* quetiapine, *RIS* risperidone, *ARP* aripiprazole, *DAI-30* Drug Attitude Inventory-30.

**Table 2:** **Monitoring sheet of “warning signs” and medication readministration criteria**

**Level 0 (Use of medication)**	**Level 1 (Slightly unfavorable)**	**Level 2 (Danger! Need for medication readministration)**
(1) Sleep duration 7–8 h	(1) Sleep duration 2–3 h	(1) Day-night reversal (No sleep)
(2) Auditory hallucinations are present, but not bothersome.	(2) Imagined voice occupies the person’s thoughts.	(2) The person suffers from auditory hallucinations involving multiple persons conversing with each other, and speaks to himself/herself.
(3) Visual hallucinations of ambiguous images persist for 10–20 s, but are not bothersome.	(3) The person sees human faces as visual hallucinations.	(3) The person sees faces as hallucinations all the time, which disturbs his/her everyday life.
(4) The person looks calm and smiles during conversation.	(4) The person uses awkward facial expressions and is unable to carry out conversations.	(4) The person looks scared, and is unable to carry out any meaningful conversation.
(5) The person eats most meals without stopping.	(5) Mealtime is often interrupted by visual hallucinations.	(5) The amount of food the person eats decreases due to visual hallucinations.
(6) The person bathes and changes clothes every day.	(6) The person bathes and changes clothes only every 3 days due to reasons other than cold weather.	(6) The person bathes and changes clothes once a week due to reasons other than cold weather.
(7) The person sometimes becomes frustrated.	(7) The person is frustrated all the time.	(7) The person hits things, such as a bed, using an object.
(8) The person is not bothered by noise.	(8) The person becomes sensitive to sound, and this startles and wakes up the person during sleep.	(8) The person always feels that he/she is under attack from sounds in the environment.
(9) The person can lead a peaceful and quiet life.	(9) The person feels restless and exhausted due to auditory hallucinations.	(9) The person is always walking around inside or outside his/her room.
(10) The person focuses on and participates in the program.	(10) The person has difficulties focusing on the program.	(10) The person drops out of the program.
(11) The person usually has a relaxing time in his/her room.	(11) The person is unable to stay in his/her room.	(11) The person is unable to sit still.
Medication readministration criteria	More than four choices	One or more choices

Changes in patients’ attitudes toward medication may influence other medical treatment programs conducted during MDP implementation, for example, a program for achieving self-management of medication. In this program, patients are encouraged by nurses to take a medicine voluntarily (only hypnotics in the intervention group), considering the necessity, action, and side effects of the drugs. To verify this point, a total of 17 subjects with schizophrenia whose basic attributes matched those of the subjects in the intervention group were used as a non-intervention group (Table 1). Experienced psychiatrists diagnosed these subjects based on DSM-IV criteria.

In conducting the program, special attention was paid to selecting the subjects because medication discontinuation, which is generally not done under usual treatment, was to be performed for subjects whose symptoms were stabilized by the effects of antipsychotic drugs, although the subjects strongly wished to discontinue medication. From an ethical perspective, we carefully examined not only the risks for prolonged hospitalization and recidivism due to aggravation of the disease but also those for poor therapeutic responsiveness resulting from changes in biological functions, including atrophy of the brain. Subjects in the intervention group hoped strongly to interrupt their medication, even after they were given information about the disadvantages of medication interruption during the disease education portion of the program. In addition, they were considered to have a high risk of interrupting medication after hospital discharge. We believe that improving medication adherence is essential to preventing recidivism, which is the purpose of the MTSA. We also believe that preventing recidivism cannot be accomplished by methods other than the MDP. This program was fully examined and approved by the ethics committee of the Department of Forensic Psychiatry with external psychiatrists. During the program, medical conditions of the subjects undergoing medication discontinuation were regularly and carefully assessed by the multidisciplinary team. These team members underwent specialized training to estimate psychiatric symptoms as appropriately as possible, and the propriety of continuing the program was examined in conference with all medical personnel to minimize bias as well as ensure that the program was executed safely. In addition, the details and implementation status of the program were described in the subjects’ treatment status records, which were regularly submitted to the court, to seek judicial comments. Subjects were informed in writing of the process for conducting the program and medication readministration. In addition, subjects were informed that they could withdraw from the MDP at any time. Written consent was obtained from all subjects.

### Procedure

#### MDP implementation guidance

##### MDP implementation period

The MDP was conducted in the early stages of the recovery phase, in which medical conditions were stabilized by antipsychotic drug therapy, and basic disease education and psychoeducation on antipsychotic drugs were provided (Figure 3). First, standard disease education was conducted using a textbook that was created by our multidisciplinary team based on the *Textbook of Psychoeducation for Patients to Understand Schizophrenia* [17]. Changes in psychiatric symptoms expected to occur by initiation of the MDP were examined individually, and warning signs of symptom aggravation were confirmed with the MDP participants. Participants were assessed as having a good understanding of this point as they appeared convinced by the examination outcome. Details of “warning signs” are shown in Table 2. We created a monitoring sheet of “warning signs” and referred to it as the “crisis plan”. A “crisis plan” was created for almost all subjects in the MTSA ward in Japan. In the “crisis plan”, the symptoms (“warning signs”) that appear in a step-by-step fashion with aggravation of the disease were indicated, and the coping skills and surrounding support methods for “warning signs” were provided. Creating a “crisis plan” is expected to facilitate the subjects’ understanding of the characteristics of and treatments for the disease and to raise the ability to self-manage the disease. Details of the “crisis plan” have been reported previously [18]. The medication readministration criteria were determined by the multidisciplinary team based on their clinical experience, as no preceding study existed. Members of the team conferred in detail regarding readministration criteria, as if the criteria are too loose, symptoms may be aggravated so much that subjects harm others or themselves, and if criteria are too strict, subjects will not have the opportunity to increase their awareness of the effects of psychiatric drugs. When the medication readministration criteria were decided, patients’ opinions were also taken into account. While subjects originally stated that they did not require medication, one subject believed their symptoms were manageable without medication, if they had four or less symptoms categorized at level 1 according to the medication readministration criteria. Coincidentally, the draft of the criteria, based on the clinical experience of the multidisciplinary team, coincided with subject comments. Subsequently, this evaluation was also used for making a decision. After consideration of these points, medication readministration criteria were selected. The basic attitude of the MDP multidisciplinary team was not to perform the MDP forcibly in a way that would invoke conflict between physicians and MDP subjects who did not recognize the need for medication and treatment but instead to examine the need for medication and the risks of recurrence in a safe way together with the subjects through the program.

##### Medication discontinuation period

After the discontinuation of medication, the presence/absence and degrees of “warning signs” and changes in psychiatric symptoms were confirmed every day using monitoring sheets between the multidisciplinary team and MDP participants, so they could share the fact that the subjects had reached an acceptable level for medication read ministration criteria. After initiation of the program, subjects were allowed to take sleep-inducing drugs, but not antipsychotic drugs. However, if subjects wished to take antipsychotic drugs, they were permitted to take them, and the MDP proceeded to the Medication readministration period at that point. In conducting the MDP, antipsychotic medication was decreased at a rate of chlorpromazine-equivalent 50 mg per week and discontinued to reduce the risk of withdrawal syndrome.

##### Medication readministration period

Even after antipsychotic drugs were restarted by reaching the medication readministration criteria, subjective and objective changes in psychiatric symptoms were confirmed using the monitoring sheet, and the need for medication and risks of recurrence were examined between the multidisciplinary team and MDP participants to promote their awareness of pharmaceutical benefits.

#### Evaluation methods

##### Patients’ characteristics

Factors that are considered to affect medication adherence in the early stages of the recovery phase before conducting the MDP were compared between the intervention and non-intervention groups.

##### Medication adherence

The DAI-30 was conducted, and the utility of the MDP was examined based on differences between DAI-30 scores before and after the implementation of the MDP in the intervention group (*n* = 7). To examine the long-term effects of the MDP, the DAI-30 was conducted in the intervention group after they had been shifted to the rehabilitation phase (6–14 months after the restart of medication). To examine changes in factors affecting medication adherence, items of the DAI-30 were categorized into three subscales: “awareness of the need for medication”, “awareness of the effects of psychiatric drugs”, and “impression of medication” (Table 3).

**Table 3:** **Subscales of the Drug Attitude Inventory-30 (DAI-30)**

**Subscales of the DAI-30**	**Questions**
Awareness of the need for medication	1. I don’t need to take medication once I feel better.
	4. Even when I am not in hospital, I need medication regularly.
	5. If I take medication, it’s only because of pressure from other people.
	8. I take medications of my own free will.
	13. I take medication only when I feel ill.
	17. I know better than the doctors when to stop taking medication.
	22. I should keep taking medication even if I feel well.
	24. It is up to the doctor to decide when I should stop taking medication.
	27. I am given medication to control behavior that other people (not myself) don’t like.
	30. By staying on medication, I can prevent myself getting sick.
Awareness of the effects of psychiatric drugs	2. For me, the good things about medication outweigh the bad.
	6. I am more aware of what I am doing, of what is going on around me, when I am on medication.
	9. Medications make me feel more relaxed.
	10. I am no different on or off medication.
	15. I get along better with people when I am on medication.
	18. I feel more normal on medication.
	21. My thoughts are clearer on medication.
	23. Taking medication will prevent me from having a breakdown.
	26. I am happier and feel better when I am taking medication.
	29. I am in better control of myself when taking medication.
Impression of medication	3. I feel strange, “doped up”, on medication.
	7. Taking medication will do me no harm.
	11. The unpleasant effects of medication are always present.
	12. Medication makes me feel tired and sluggish.
	14. Medication is slow-acting poison.
	16. I can’t concentrate on anything when I am taking medication.
	19. I would rather be ill than take medication.
	20. It is unnatural for my mind and body to be controlled by medication.
	25. Things that I could do easily are much more difficult when I am on medication.
	28. I can’t relax on medication.

##### Effect of excluding other programs

To confirm whether other programs were affecting the DAI-30 scores, we compared the DAI-30 scores in the same period in the non-intervention group (*n* = 17). For the non-intervention group, the initial evaluation was conducted in the early stages of the recovery phase and a second evaluation was conducted 30.3 ± 7.7 days later.

##### Changes in psychiatric symptoms after the implementation of the MDP

The Brief Psychiatric Rating Scale (BPRS) was used for continuous evaluation of the levels of aggravation resulting from medication discontinuation in the intervention group.

##### Statistics

The two-sample *t*-test was conducted to compare DAI-30 scores before and after the implementation of the MDP in the intervention group and in the same period of non-intervention group at the 0.05 significance level. Mann-Whitney *U* test and Fisher’s exact test were conducted to compare patients’ characteristics between the intervention and non-intervention groups at the 0.05 significance level. SPSS PASW Statistics 18 (SPSS Co., Ltd., Tokyo, Japan) was used for statistical analysis.

## Results

Patients’ characteristics in the intervention and non-intervention groups are shown in Table 4. Although the significance of the statistical processing is poor, as the sample size is so small, we compared characteristics that were considered to affect medication adherence between the two groups. No significant differences were observed by the Mann-Whitney *U* test and Fisher’s exact test (data not shown).

**Table 4:** **Factors measured in the early stages of the recovery phase**

	**Intervention group (** ***n*** **= 7)**	**Non-intervention group (** ***n*** **= 17)**
Gender	Male: 6, Female: 1	Male: 15, Female: 2
Age	45.3 ± 9.3	43.1 ± 11.4
Full-scale IQ	91.7 ± 14.7	92.8 ± 15.1
Years of education	15.4 ± 2.5	14.4 ± 2.6
DUP (year)	7.5 ± 7.5	3.2 ± 4.6
Treatment duration (year)	3.0 ± 4.5	9.8 ± 12.6
GAF	52.4 ± 7.1	53.1 ± 10.2
Family members living together	Present: 4	Present: 14
	Absent: 3	Absent: 3
Main antipsychotic drug	OLZ: 5	OLZ: 10
		RIS: 3
	RIS: 2	QTP: 3
		ARP: 1
CP equivalents (mg)	414.3 ± 211.6	698.9 ± 498.2
Self-discontinuation of antipsychotic medication	Present: 4	Present: 14
	Absent: 3	Absent: 3

*DUP* duration of untreated psychosis, *GAF* Global Assessment of Functioning, *CP* chlorpromazine, *OLZ* olanzapine, *RIS* risperidone, *QTP* quetiapine, *ARP* aripiprazole.

**Figure 4:**
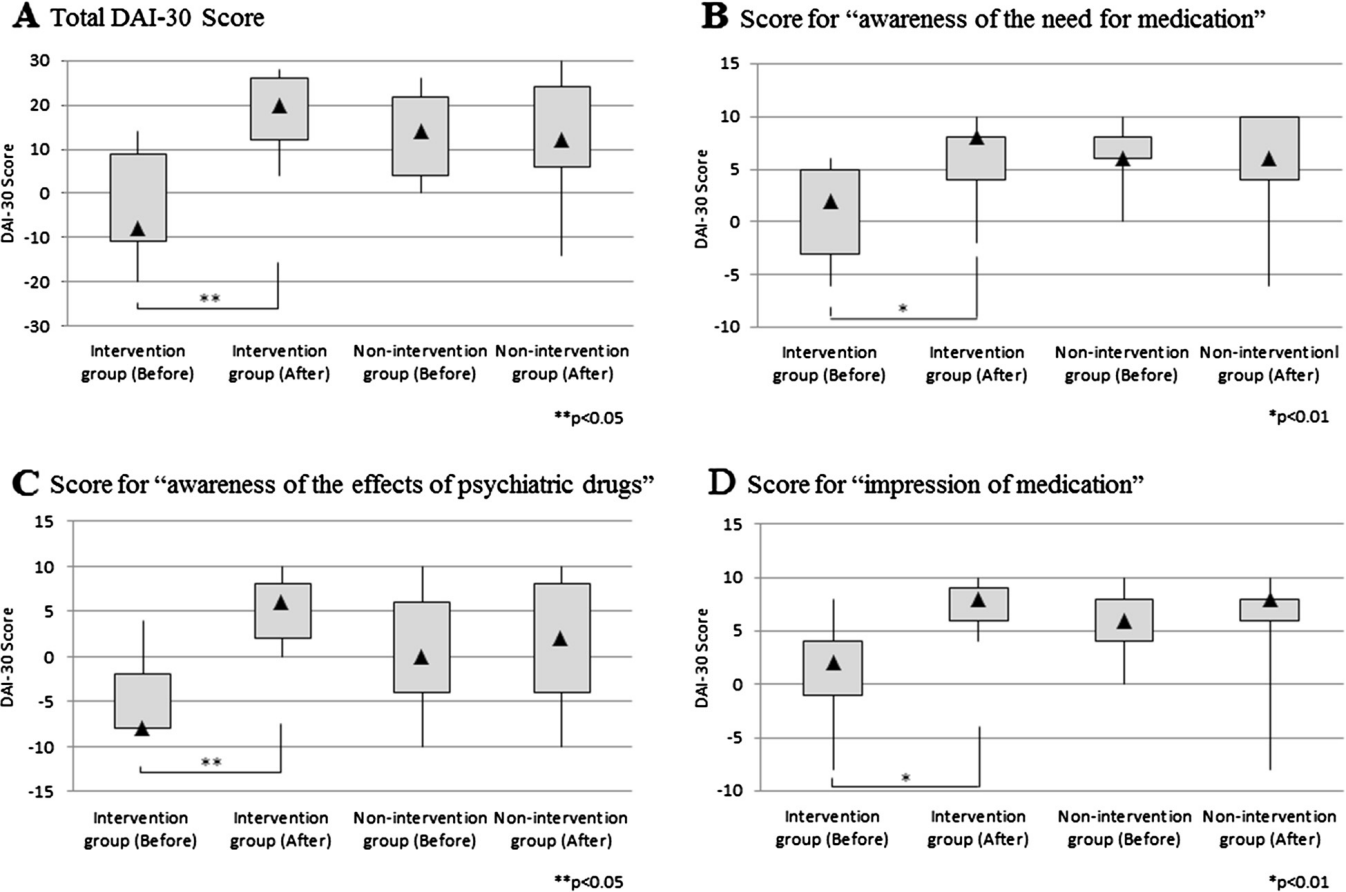
**Changes in the Drug Attitude Inventory-30 (DAI-30) score from the Medication Discontinuation Program (MDP) intervention group (** ***n*** **= 7) and non-intervention group (** ***n*** **= 17).** The DAI-30 was conducted before and after implementation of the MDP in the intervention group. In the non-intervention group, initial evaluation was conducted in the early stages of the recovery phase and a second evaluation was conducted 30.3 ± 7.7 days later. The DAI-30 scores are shown by box-and-whisker plots. Black triangles indicate the median value in each group. In the intervention group, the mean scores of the DAI-30 measured before and after the MDP are as follows: **(A)** Total: −2.6 ± 13.2 and 18.3 ± 9.2 (*P* = 0.002); **(B)** “awareness of the need for medication”: 0.9 ± 4.9 and 5.7 ± 4.2 (*P* = 0.015); **(C)** “awareness of the effects of psychiatric drugs”: −4.6 ± 4.7 and 5.1 ± 3.8 (*P* = 0.002); **(D)** “impression of medication”: 1.1 ± 5.1 and 7.4 ± 2.2 (*P* = 0.014). Before: before the MDP, After: after the MDP, Initial: initial evaluation, Second: second evaluation. * *P* < 0.01, ** *P* < 0.05 by the two-sample *t*-test.

Table 5 shows changes in the DAI-30 score in the intervention group. The total DAI-30 score significantly increased after the completion of the MDP in the intervention group (*P* = 0.002) (Figure 4A). In this group, the mean total score of the DAI-30 was 18.3 ± 9.2 after completion of the MDP and 19.9 ± 8.5 after shifting to the rehabilitation phase. Although no significant elevation was observed in DAI-30 score after shifting to the rehabilitation phase, the score was similar to or slightly higher than that measured after completion of the MDP. No significant differences were observed in the total DAI-30 score between the initial evaluation and after evaluation in the non-intervention group (Table 6).

**Table 5:** **Changes in Drug Attitude Inventory-30 (DAI-30) score in the intervention group**

	**Before**	**After**	**After shifting to the recovery phase**	**Period of time before readministering antipsychotic drugs**
Case 1	−14	4	6 (10 months after completion of the MDP)	2 days
Case 3	−8	10	16 (6 months after)	15 days
Case 4	−8	26	26 (8 months after)	9 days
Case 5	14	20	20 (14 months after)	14 days
Case 6	8	28	30 (6 months after)	15 days
Case 7	10	26	27 (7 months after)	36 days
Case 8	−20	14	14 (6 months after)	27 days
Mean ± SD	−2.6 ± 13.2	18.3 ± 9.2	19.9 ± 8.5	

Before means before the Medication Discontinuation Program (MDP); After means after the MDP.

**Table 6:** **Changes in Drug Attitude Inventory-30 (DAI-30) scores in the non-intervention group**

	**Initial**	**Second**
Case a	0	0
Case b	14	6
Case c	4	2
Case d	24	24
Case e	10	0
Case f	4	12
Case g	18	12
Case h	10	28
Case i	4	8
Case j	2	10
Case k	26	30
Case l	26	20
Case m	22	−14
Case n	14	28
Case o	10	12
Case p	26	28
Case q	20	22
Mean ± SD	13.8 ± 9.1	13.4 ± 12.5

Initial means initial evaluation; Second means second evaluation.

Significant elevations after completion of the MDP were also observed in the scores for all subscales of the DAI-30, that is, “awareness of the need for medication,” “awareness of the effects of psychiatric drugs,” and “impression of medication” (*P* = 0.015, *P* = 0.002, and *P* = 0.014, respectively); however, there were no elevations in the non-intervention group (Figure 4B–D, respectively).

The period of time before readministering antipsychotic drugs was 2 days at the earliest and 36 days at the latest. After the readministration of antipsychotic drugs, BPRS scores rapidly decreased to the same level or lower as scores observed at the initiation of the MDP in all subjects in the intervention group (Table 7).

**Table 7:** **Changes in the Brief Psychiatric Rating Scale (BPRS) scores in the intervention group**

**Case**	**Total BPRS score before discontinuing medication**	**BPRS subscale items**		**Total BPRS score at readministration of medication**	**BPRS subscale items**		**Total BPRS score 2 weeks after readministering medication**	**BPRS subscale items**	
1	38	Positive	29	41	Positive	30	38	Positive	29
		Negative	3		Negative	3		Negative	3
		Neurotic	6		Neurotic	8		Neurotic	6
3	26	Positive	14	41	Positive	23	26	Positive	14
					Negative	6		Negative	6
		Negative	6						
		Neurotic	6		Neurotic	12		Neurotic	6
4	31	Positive	16	40	Positive	22	31	Positive	16
		Negative	6		Negative	6		Negative	6
		Neurotic	9		Neurotic	12		Neurotic	9
5	24	Positive	11	29	Positive	19	23	Positive	11
		Negative	5		Negative	4		Negative	5
		Neurotic	8		Neurotic	6			
								Neurotic	7
6	20	Positive	11	31	Positive	18	20	Positive	11
		Negative	5					Negative	5
		Neurotic	4		Negative	6			
					Neurotic	7			
								Neurotic	4
7	29	Positive	17	35	Positive	21	26	Positive	14
		Negative	4		Negative	4		Negative	4
		Neurotic	8		Neurotic	10		Neurotic	8
8	25	Positive	13	28	Positive	16	24	Positive	13
		Negative	5		Negative	3		Negative	4
		Neurotic	7		Neurotic	9		Neurotic	7
2	40	Positive	24	42	Positive	25	43	Positive	25
		Negative	9	3 months after MDP implementation	Negative	9	1 year after MDP implementation	Negative	10
		Neurotic	7		Neurotic	8		Neurotic	8

Positive means positive symptoms; Negative means negative symptoms; Neurotic means neurotic symptoms.

## Discussion

### Utility of the MDP

In the intervention group, the total score on the DAI-30 and scores for the subscales significantly improved after the MDP. These results suggest that the MDP may help improve medication adherence. Furthermore, score reductions were not observed even approximately 6–14 months after the program, indicating the possibility of long-term effects of the MDP. However, no significant elevations of DAI-30 scores were observed in the non-intervention group during the study, suggesting that other treatment programs conducted during MDP implementation or after completion of the MDP do not affect DAI-30 score. This result also might indicate that the DAI-30 scores were relatively stable over time in patients who were in non-acute phases of schizophrenia.

In the intervention group, the total score for DAI-30 and those for the subscales significantly improved, possibly because the MDP had multidirectional and stimulatory effects on each factor relating to the improvement of medication adherence. Success in the establishment of a treatment alliance, which was facilitated by conducting a team-based program including the subject based on individual needs, may also have contributed to the improvement in DAI-30 scores.

Morken et al. reported that one-sided education and interventions by medical providers are not effective in improving medication adherence, so a patient’s proactive involvement in interventions is needed [19]. Furthermore, Dolder et al. reported that the improvement in adherence was seen in interventions using a combination of educational, behavioral, and affective strategies. They also described that longer interventions and an alliance with therapists appeared important for successful outcomes [20].

The MDP is also an intervention using a combination of educational, behavioral, and affective strategies in which patients can be proactively involved. Furthermore, this program is a long-term intervention that lasts for a maximum of 6 months because the program involves disease education and monitoring of “warning signs” before, during, and after the discontinuation of medication. Strong and trusting relationships that serve as a basis of treatment alliance may have been structured during this long-term intervention. These factors may explain how the MDP contributed to improving medication adherence.

### A withdrawal case

Case 2, who withdrew from the MDP, had a high IQ but had marked cognitive dysfunction. He did not reject pharmacotherapy and psychosocial treatment provided by the multidisciplinary team but had a passive attitude toward it, so he could not deepen his insight into the disease. However, he wished to participate only in the MDP and showed an active attitude toward it.

Amador et al. reported that hallucinations, loss of pleasure, and reduced sociality are more easily recognized than delusions and thought disturbances by patients with schizophrenia, and indicated that whether or not patients can acquire insight is associated with symptoms of schizophrenia [21].

Case 2 did not present hallucinations, loss of pleasure, and reduced sociality but had delusions and thought disturbances, which are considered difficult for patients to become aware of. Furthermore, case 2 showed marked alterations in his personality, such as cynical, arrogant, and unnatural behaviors. Therefore, he was unable to notice changes in his condition after discontinuation of antipsychotic drugs, and it was difficult to evaluate him objectively. These results might suggest that, for patients with barely noticeable hallucinations and main clinical features of delusions and thought disturbances, and those with chronic schizophrenia who present marked alterations in personality, the MDP is not suitable. Further study should be performed to examine this point in a larger number of subjects.

### Limitations

This study has several limitations. First, the sample size was small. The MDP should be performed for patients who meet the strict criteria: those who strongly deny their disease, refuse to take medication, and wish to discontinue medication, patients who can verbally describe changes in their medical conditions, have a certain level of IQ and are not likely to hide their medical condition. However, the availability of such patients was extremely limited. Furthermore, we cannot perform a clinical study that may interfere with healing, as we treat patients in the forensic ward based on the national policy, and are obliged to rehabilitate patients promptly. Performing the program for patients treated under the MTSA requires much effort and remains a major issue that needs to be examined in the future.

Second, as an evaluation scale, we used DAI-30, which is a self-completed questionnaire. The change in patients’ attitudes to medication is a crucial point in this study. Therefore, an objective evaluation scale should be used. Unfortunately, such an evaluation scale does not exist at present. After the completion of the MDP, the multidisciplinary team actually observed an improvement in medication adherence, acceptance of the disease, and reduction in the resistance to medical treatment in the subjects. Furthermore, to evaluate the effects of the program, rating scales and the assessment of health status during hospitalization are insufficient. Long-term follow-up based on medication adherence, changes in health status, and living status after hospital discharge is needed. We are currently performing a follow-up investigation involving subjects who completed hospitalized treatment in our department and shifted to outpatient treatment.

Third, in our study, we used medication readministration criteria that were not validated objectively. Subjects were carefully selected and medical conditions of subjects undergoing medication discontinuation were regularly and carefully assessed by the multidisciplinary team. Therefore, while we may be able to execute a program safely using these medication readministration criteria, this does not necessarily ensure the complete reliability of the program. We cannot exclude the possibility of discrepancies as the safety of the MDP has not been affirmed. Further study is essential in determining the best medication readministration criteria.

Although this study has some limitations, we think the MDP might become a useful treatment program to improve medication adherence, an important issue in the treatment of schizophrenia. The BPRS scores worsened after medication discontinuation, although they recovered promptly after medication readministration. These results suggest that this program was executed safely. A randomized case-control study will be needed to prove the validity of the MDP in the future. However, as mentioned above, there are several difficulties in using such a research design at our ward. The selection of the control group is particularly difficult. Some consideration is needed to address this point. For example, one solution may be to measure DAI-30 for a certain fixed period in subjects in the intervention group before MDP intervention and use these DAI-30 scores as control data. After that, subjects would undergo the same intervention reported in this paper. This design might be considered a quasi-case control study. We expect that the MDP will be tried not only in the judicial ward but many general psychiatric care institutions to clarify both its usefulness and limitations. In the process, MDP will become further refined. At that time, MDP may be applicable not only to medical care for mentally ill offenders but also to the treatment of schizophrenia.

## Conclusions

We conducted the MDP that was developed to increase awareness of the effects of psychiatric drugs and improve medication adherence. Our study suggests that the MDP has the possibility of improving medication adherence and might have multidirectional and stimulatory effects on each factor relating to this improvement. It was also suggested that the effects of the MDP may be maintained over a long duration. However, this is a pilot study and has some limitations. Further studies should be performed to replicate the present finding in a larger number of subjects.

## Abbreviations

BPRS: Brief Psychiatric Rating Scale
DAI-30: Drug Attitude Inventory-30
DSM-IV: Diagnostic and Statistical Manual of Mental Disorders - Fourth Edition
GAF: Global Assessment of Functioning
MDP: Medication Discontinuation Program
MTSA: Medical Treatment and Supervision Act
WAIS-III: Wechsler Adult Intelligence Scale - Third Edition
